# Moderate-to-Severe Malnutrition Identified by the Controlling Nutritional Status (CONUT) Score Is Significantly Associated with Treatment Failure of Periprosthetic Joint Infection

**DOI:** 10.3390/nu14204433

**Published:** 2022-10-21

**Authors:** Zhuo Li, Zulipikaer Maimaiti, Zhi-Yuan Li, Jun Fu, Li-Bo Hao, Chi Xu, Ji-Ying Chen

**Affiliations:** 1 School of Medicine, Nankai University, Tianjin 300071, China; 2 Department of Orthopedics, The First Medical Center, Chinese PLA General Hospital, Beijing 100853, China; 3 Department of Orthopedics, The Fourth Medical Center, Chinese PLA General Hospital, Beijing 100048, China

**Keywords:** periprosthetic joint infection, malnutrition, Controlling Nutritional Status, outcome

## Abstract

The prevalence and role of malnutrition in periprosthetic joint infection (PJI) remain unclear. This study aimed to use measurable nutritional screening tools to assess the prevalence of malnutrition in PJI patients during two-stage exchange arthroplasty and to explore the association between malnutrition and treatment failure. Our study retrospectively included 183 PJI cases who underwent 1st stage exchange arthroplasty and had available nutritional parameters, of which 167 proceeded with 2nd stage reimplantation. The recently proposed Musculoskeletal Infection Society (MSIS) Outcome Reporting Tool was used to determine clinical outcomes. The Controlling Nutritional Status (CONUT), Nutritional Risk Index (NRI), and Naples Prognostic Score (NPS) were used to identify malnutrition at 1st and 2nd stage exchange, respectively. Multivariate logistic regression analyses were performed to determine the association between malnutrition and treatment failure. Restricted cubic spline models were further used to explore the dose–response association. Additionally, risk factors for moderate-to-severe malnutrition were evaluated. Malnourished patients identified by CONUT, NPS, and NRI accounted for 48.1% (88/183), 98.9% (181/183), and 55.7% (102/183) of patients at 1st stage, and 9.0% (15/167), 41.9% (70/167), and 43.1% (72/167) at 2nd stage, indicating a significant improvement in nutritional status. We found that poorer nutritional status was a predictor of treatment failure, with CONUT performing best as a predictive tool. Moderate-to-severe malnutrition at 1st stage identified by CONUT was significantly related to treatment failure directly caused by PJI (odds ratio [OR] = 5.86), while the OR was raised to 12.15 at 2nd stage (OR = 12.15). The linear dose–response associations between them were also confirmed (P for nonlinearity at both 1st and 2nd stage > 0.05). As for total treatment failure, moderate-to-severe malnutrition as determined by CONUT was associated with a 1.96-fold and 8.99-fold elevated risk at the 1st and 2nd stages, respectively. Age ≥ 68 years (OR = 5.35) and an increased number of previous surgeries (OR = 2.04) may be risk factors for moderate-to-severe malnutrition. Overall, the prevalence of malnutrition in PJI patients is very high. Given the strong association between moderate-to-severe malnutrition identified by CONUT and PJI treatment failure, COUNT could be a promising tool to evaluate the nutritional status of PJI patients to optimize treatment outcomes.

## 1. Introduction

Periprosthetic joint infection (PJI) is the most devastating complication after total joint arthroplasty (TJA). The number of PJIs is estimated to grow exponentially in the next decade due to the aging population and the dramatic increase of TJA [1,2,3]. The current standard management protocol for chronic PJI remains a two-stage exchange arthroplasty; however, it is exceptionally technically demanding, with a concerning failure rate [4,5]. Additionally, PJI treatment failure is not only associated with considerable medical costs but is also accompanied by higher mortality than some common cancers [6,7]. Further efforts to improve the prognosis of patients with PJI are urgently needed [8].

Malnutrition generally implies undernutrition and refers to deviations from adequate and optimal nutritional status [9]. As a modifiable patient-related risk factor, it is associated with poor outcomes in various diseases [10,11]. To date, several studies have also preliminarily investigated the effects of malnutrition on clinical outcomes following primary TJA, reporting impaired wound healing and greater susceptibility to infection, as well as an increase in other complications [12,13,14,15,16]. Although not fully elucidated, potential mechanisms included a disrupted immune system and physical barrier due to reduced lymphocyte and collagen synthesis [17]. Of more significant concern is the high prevalence of malnutrition in the TJA population, ranging from 8.5% to 50%, suggesting that we have a considerable opportunity to improve their clinical outcomes further [18]. Recent studies have further confirmed that nutritional interventions for TJA patients could provide postoperative benefits [19,20,21]. 

Extensive studies have reported a synergistic relationship between malnutrition and infection. The main reason for the increased susceptibility to infection in malnourished hosts may be the extensive compromise of immune function, with potential mechanisms including impaired mucosal and skin barrier function, reduced macrophage numbers and phagocytosis, decreased natural killer cell activity, and defects in the adaptive immune system, represented by T cells [22]. The immune response stimulated by infection increases the demand for energy, leading to a vicious cycle of poor nutritional status and severe infection [23]. Overall, the relationship between malnutrition, immunosuppression, and infection, as one of the pathophysiological bases of infectious diseases, is complex and profound. 

More critically, in clinical settings, pathological conditions such as chronic infections and cancer have been documented to induce a higher prevalence of malnutrition, resulting in worse clinical outcomes [24,25,26,27]. Given the above facts, the potential significance of malnutrition for PJI, a chronic wasting infectious disease, may be of even greater concern. However, reports of malnutrition in patients with PJI are extremely scarce. A study by Zajonz D et al. [28] demonstrated the presence of reduced albumin and total protein levels in PJI. Based on laboratory parameters such as serum albumin, two studies have revealed a 2–3 times higher incidence of malnutrition in PJI patients than in those with aseptic loosening [16,29]. These studies remain inadequate to guide clinical practice due to small sample sizes, inconsistent nutritional assessments, and lack of exploration of the impact on clinical outcomes. 

Previous nutritional assessments in arthroplasty were primarily based on individual blood parameters [17,18,30]. Nevertheless, their value as a surrogate of malnutrition to predict wound complications after TJA has recently been questioned [31]. As of today, nutritional assessment can be achieved effectively with different measurable immunonutritional screening tools (INST), such as Controlling Nutritional Status (CONUT) [32], Nutritional Risk Index (NRI) [33], and Naples Prognostic Score (NPS) [34]. These tools have demonstrated promising potential in the nutritional and prognostic assessment of various diseases [35,36,37]. Another major advantage of them is the availability and feasibility of the included parameters in many hospitals. The application of these INSTs to the field of arthroplasty or PJI may provide more valuable and generalizable information. 

Therefore, this study aimed to determine the prevalence of malnutrition identified by INSTs in PJI patients and to explore the association between malnutrition and treatment outcomes. Meanwhile, it could provide further clinical insights into malnutrition in infectious diseases. 

## 2. Materials and Methods

### 2.1. Study Population

**Figure 1:**
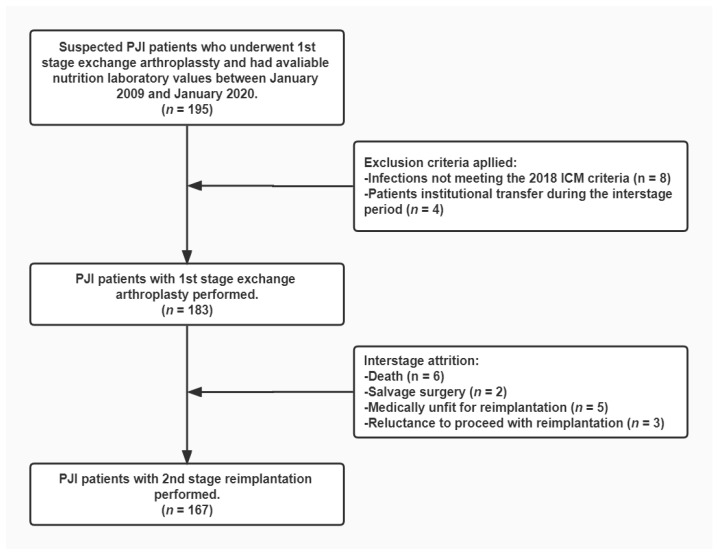
This flowchart demonstrates how patients were identified for inclusion in the final cohort. PJI, periprosthetic joint infection; ICM, International Consensus Meeting.

After Institutional Review Board approval, we retrospectively reviewed 195 suspected PJI cases who received 1st stage exchange arthroplasty at our institution and had available nutritional parameters between January 2009 and January 2020. The required nutritional laboratory tests included routine blood and biochemical tests. We excluded 12 patients, including 8 patients who did not meet the International Consensus Meeting (ICM) 2018 criteria [38] and 4 patients with institutional transfer during the interstage period of the two-stage exchange. Figure 1 depicts the enrollment of the study cohort, with a total of 183 PJI cases included in the final study. 

### 2.2. Two-Stage Exchange Technique

Two-stage exchange arthroplasty is the standard regimen for chronic PJI at our institution. Institution-based surgical techniques performed by high-volume surgeons were used for all patients. Briefly, the 1st stage exchange consists of removal of the infected component, thorough debridement, and placement of an antibiotic-impregnated spacer. Multiple microbiological cultures guide the systematic administration of antibiotics. After 6–12 weeks of antibiotic therapy, patients are evaluated comprehensively for the timing of 2nd stage reimplantation, including laboratory tests and clinical signs. If the infection is considered to be eradicated, a redebridement is performed and a new prosthesis is reinserted. In our study, 167 patients proceeded with 2nd stage reimplantation. Interstage attrition occurred in 16 patients. Of them, five patients were assessed as medically unfit for reimplantation, six died, three were reluctant to proceed with reimplantation, and two underwent salvage surgery (Figure 1). 

### 2.3. Clinical Data Extraction and Nutritional Status Assessment

Two investigators independently reviewed the patients’ medical records of 1st and 2nd stage exchange. Nutrition-related parameters were extracted, including laboratory values of serum albumin, lymphocytes, neutrophils, monocytes, and total cholesterol. Based on the availability of the parameters and their essential value in the diagnosis and treatment of PJI, c-reactive protein (CRP) and erythrocyte sedimentation rate (ESR) were also obtained to reflect the systemic inflammatory response [39,40]. In addition, demographic details, microbiological data, and comorbidities were summarized. 

The CONUT, NPS, and NRI were used to assess the nutritional status of PJI patients. The CONUT score was calculated from total cholesterol, lymphocytes, and serum albumin, with 0–1, 2–4, and >5 representing normal, mild, and moderate-to-severe malnutrition, respectively [32]. The NPS score was determined by neutrophil: lymphocyte ratio, lymphocyte: monocyte ratio, serum albumin, and total cholesterol level. Scores of 1–2 and 3–4 were considered mild or moderate-to-severe malnutrition, respectively [34]. The NRI was calculated as 1.519 × serum albumin +41.7 × (current body weight [kg]/usual body weight [kg]) [33]. The ideal weight was usually used instead of the usual weight, defined as height (cm)-100-([height (cm)-150]/2.5) in women and height (cm)-100-([height (cm)-150]/4) in men. NRI ≥ 100 represented no nutritional risk, 97.5 ≤ NRI < 100 represented mild risk, and NRI < 97.5 represented moderate-to-severe risk. 

### 2.4. Clinical Outcome Assessment

The mean follow-up time was 7.56 (median, 7.19) years after resection. The recently proposed Musculoskeletal Infection Society (MSIS) Outcome Reporting Tool was used to determine clinical outcomes [41]. This tool allows for more accurate classification of outcomes by categorizing them into four tiers. The MSIS recommends setting the 1st stage of the two-stage exchange as the starting point for assessment. To evaluate the impact of malnutrition at each stage, we placed the 1st and 2nd stage exchange as the starting point of our study, respectively. Infection control with or without antibiotic therapy was considered Tier 2 or Tier 1. Aseptic and septic revisions performed more than one year postoperatively were assessed as Tiers 3A and 3B. In comparison, aseptic and septic revisions performed less than one year postoperatively were considered Tiers 3C and 3D, respectively. Tiers 3E and 3F represented salvage surgery and spacer retention, respectively. Tiers 4A and 4B indicated death within one year and one year after surgery, respectively. According to Borsinger et al. [42], Tiers 1 and 2 were considered treatment successes, while Tiers 3 and 4 were regarded as total treatment failures. Total treatment failure was further divided into two categories, with failure directly related to PJI (Tiers 3B, 3C, 3E, 3F, 4A) and failure caused by secondary causes (Tiers 3A, 4B). The relationships between malnutrition and two types of treatment failure, those directly related to PJI and total treatment failure, were explored separately. 

### 2.5. Statistical Analysis

Values were presented as mean ± SD or number of subjects (percentage). Differences in continuous variables between groups were compared using independent-samples *t*-tests. Categorical variables were compared using chi-square tests and Fisher exact tests. Venn diagrams depicted the relationship between malnourished patients identified by various INSTs. We also performed Pearson correlation analyses to explore the correlation of nutritional scores with indicators of systemic inflammation or other nutritional scores. 

After adjusting baseline characteristics such as age, gender, Body Mass Index (BMI), pathogens, previous surgeries, knee or hip joints, primary diagnosis, smoking, and comorbidities, the associations between malnutrition at the 1st or 2nd stage exchange and failures directly related to PJI were assessed using logistic regression analysis. We investigated the impact of mild or moderate-to-severe malnutrition on outcomes, respectively, with normal nutritional status identified by different INSTs as the reference category. Another logistic regression analysis was performed to identify risk factors of moderate-to-severe malnutrition identified by CONUT at 1st stage. The receiver operating characteristic (ROC) curve determined the optimal cutoff value of age. Multivariate analysis was performed for variables with *p* < 0.3 in univariate analysis. All logistic regression analyses reported adjusted odds ratios (ORs) and 95% confidence intervals (CIs). Additionally, after adjusting baseline characteristics, we used restricted cubic splines (RCS) with three knots to evaluate the dose–response relationship of CONUT score with treatment failure directly related to PJI. 

All statistical analyses were performed by SPSS version 20 (IBM Corp., Armonk, NY, USA) and R software version 3.8.1 (R Development Core Team, Auckland, New Zealand). *p* < 0.05 was considered to indicate statistical significance. 

## 3. Results

### 3.1. Patient Characteristics

The study included 183 PJI patients who underwent 1st stage exchange, and their baseline characteristics are presented in Table 1. Forty-seven and a half percent of the patients were male, with a mean age of (59.6 ± 13.2) years and a mean BMI of (25.0 ± 4.1) kg/m^2^. The most common causative organism and primary diagnoses were coagulase-negative staphylococci (28.4%) and osteoarthritis (28.4%). 

### 3.2. Prevalent Trends and Clinical Features of Malnutrition

**Figure 2:**
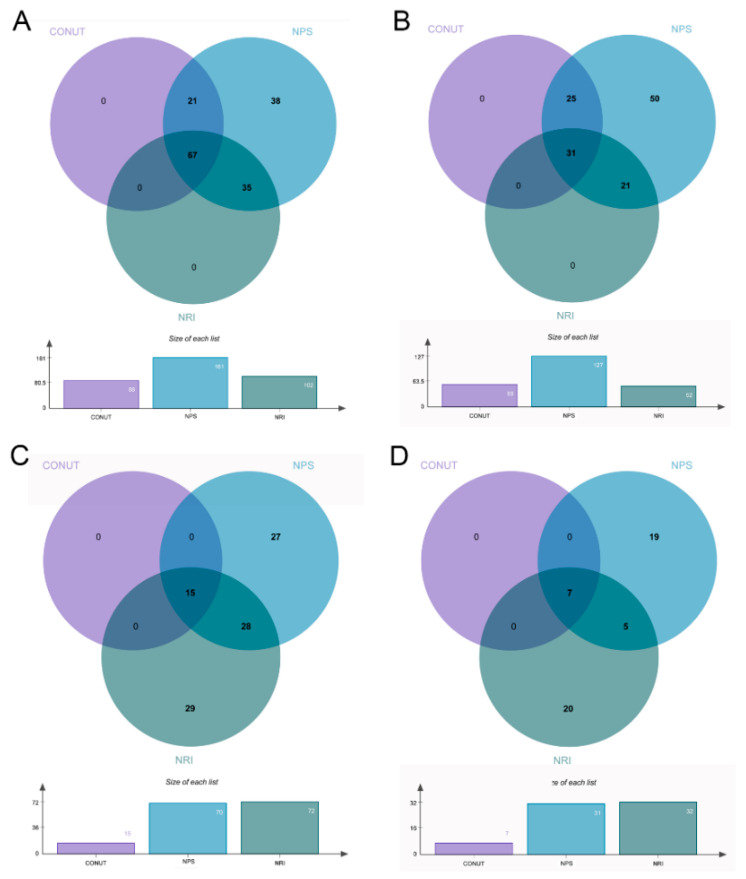
Any degree of malnutrition or moderate-to-severe malnutrition identified by different definitions at 1st stage (**A**,**B**) and 2nd stage (**C**,**D**). CONUT, Controlling Nutritional Status; NRI, Nutritional Risk Inde; NPS, Naples Prognostic Score.

As shown in Figure 2A, malnutrition identified by CONUT, NPS, and NRI accounted for 48.1% (88/183), 98.9% (181/183), and 55.7% (102/183) of patients at the 1st stage, respectively. Of them, 67 patients (36.6%) were identified by all three INSTs simultaneously. Furthermore, the percentage of moderate-to-severe malnutrition varied from 28.4% (52/183) with NRI to 69.4% (127/183) with NPS (Figure 2B). Among the 167 patients who proceeded with reimplantation, malnutrition identified by CONUT, NPS, and NRI accounted for 9.0% (15/167), 41.9% (70/167), and 43.1% (72/167), respectively (Figure 2C). The prevalence of moderate-to-severe malnutrition decreased considerably, varying from 4.2% with CONUT to 18.6% with NPS (Figure 2D). The CONUT, NPS, and NRI at the 2nd stage were all significantly improved (*p* < 0.001) compared to those at the 1st stage (Appendix A). 

Table 2 showed moderate correlations between the three INSTs (CONUT vs. NPS: r = 0.63; CONUT vs. NRI: r = −0.52; NPS vs. NRI: r = −0.53; all *p*< 0.001). In addition, correlation analysis of the three INSTs and ESR or CRP revealed a close association between poorer nutritional status and stronger inflammatory response (Table 2). 

### 3.3. Association between Malnutrition and Treatment Failure

As shown in Table 3, the failure rates directly related to PJI after the 1st and 2nd stage exchange were 17.5% and 9.6%, respectively. After adjusting for potential confounders, we assessed the association between malnutrition identified by different INSTs and these failures (Table 4). For patients with moderate-to-severe malnutrition identified by CONUT at 1st stage, their failure risk was 5.86 times higher than that of controls (95% CI, 1.39–24.67; *p* = 0.016). At the 2nd stage, the relative risk of failure in patients with moderate-to-severe malnutrition identified by CONUT or NPS increased to 12.15 (95% CI, 1.14–129.36; *p* = 0.039) or 14.39 (95% CI, 1.27–266.22; *p* = 0.033), respectively. Although statistically insignificant, moderate-to-severe malnutrition determined by NRI also tended to be associated with treatment failure directly related to PJI (OR = 3.60; 95% CI, 0.84–15.36; *p* = 0.083). Given the good predictive value of moderate-to-severe malnutrition as determined by CONUT, we further explored its association with total treatment failure. It was associated with a 1.96-fold and 8.99-fold elevated risk at the 1st and 2nd stage, respectively (Appendix B). 

**Figure 3:**
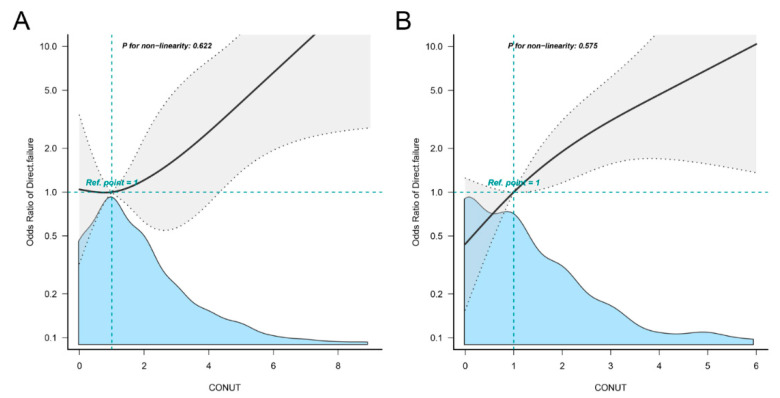
Restricted cubic spline models representing the associations of CONUT score and treatment failure directly related to PJI at 1st (**A**) or 2nd stage (**B**) exchange. Adjusted for baseline characteristics such as age, gender, Body Mass Index, pathogens, number of previous surgeries, knee or hip joints, primary diagnosis, smoking, and comorbidities. Reference group is that the value of CONUT score is 1. The gray area represents the confidence interval of the model. The blue area is the density plot of the CONUT scores. CONUT, Controlling Nutritional Status.

Figure 3 depicts the results of the multivariate-adjusted spline regression model. CONUT scores at either 1st or 2nd stage showed a linear dose–response association with treatment failure directly related to PJI (P for nonlinearity at 1st stage = 0.622; Figure 3A; P for nonlinearity at 2nd stage = 0.575; Figure 3B). 

### 3.4. Risk Factors for Moderate-to-Severe Malnutrition at 1st Stage Exchange

We further evaluated the predictors of moderate-to-severe malnutrition identified by CONUT. Univariate logistic regression analysis identified age, gender, number of previous surgeries, and primary diagnosis as potential risk factors. The ROC curve indicated that an optimal cut-off value of age was 68 years. Table 5 demonstrated the results of multivariate analysis, with age ≥68 years (OR = 5.35; 95% CI, 1.17–24.52; *p* = 0.031) and increased number of previous surgeries (OR = 2.04; 95% CI, 1.20–3.50; *p* = 0.009) as independent risk factors. Compared with osteoarthritis, a primary diagnosis of osteonecrosis of the femoral head (OR = 0.11; 95% CI, 0.01–0.87; *p* = 0.037) may be a protective factor. 

## 4. Discussion

Malnutrition has been extensively documented in the orthopedic literature to be closely associated with adverse clinical outcomes [12,13,14,15,16]. However, the paucity of relevant research in the field of PJI limits our understanding of this issue. To our knowledge, this is the first study to investigate the prevalence and impact of INST-identified malnutrition in PJI patients. Our study revealed that malnutrition was highly prevalent in PJI patients, ranging from 48.1% with CONUT to 98.9% with NPS. INST scores were significantly correlated with levels of ESR or CRP, reflecting that a poorer nutritional status may accompany a higher systemic inflammatory response. Given the high prevalence of malnutrition, the question remains as to which INST is appropriate for clinical use to identify patients with prognostically significant malnutrition. Our results showed that moderate-to-severe malnutrition identified by CONUT at either stage of the two-stage exchange was strongly associated with treatment failure. The linear dose–response associations of the CONUT score with treatment failure directly related to PJI were also confirmed. Moreover, age ≥68 years and an increased number of previous surgeries may be risk factors for moderate-to-severe malnutrition. 

Malnutrition is becoming a widely recognized comorbidity in preventing and treating musculoskeletal disorders [30]. However, nutritional assessments in previous arthroplasty-related studies were primarily based on individual parameters, such as transferrin, serum albumin, and total lymphocytes [12,13,14,15,16]. A recent study found little overlap in malnutrition identified by serum albumin or total lymphocytes alone in patients undergoing total knee arthroplasty, questioning their predictive value as proxies for malnutrition [31]. Therefore, there is an urgent need to explore these well-validated INSTs in the field of arthroplasty. Nanri et al. [43] evaluated 503 patients undergoing primary total hip arthroplasty and found that 18.9% were classified as malnourished by CONUT. Our study further revealed that the percentage of PJI patients identified as malnourished by CONUT raised to 48.1%. This incidence is alarming and even exceeds the prevalence of malnutrition in some common cancers, such as lung cancer, colorectal cancer, and esophageal cancer [44,45,46], which may be related to nutrition consumption in chronic infection. A higher proportion of malnutrition was identified by NPS or NRI, possibly due to the different nature of the INSTs. Similar variations have been reported in previous studies [36,37]. Overall, our study revealed that the prevalence of malnutrition in the PJI population is considerable. Meanwhile, the significantly improved nutritional status at the 2nd stage is of interest, suggesting that malnutrition due to infection may be reversible. 

The association between nutritional status and clinical outcomes has been confirmed in the orthopedic literature. Three meta-analyses have shown that malnutrition increases the risk of surgical site infection after TJA [47,48,49]. Additionally, malnutrition-related complications after TJA reported in the literature include cardiac arrest, pneumonia, readmission rates, reoperation rates, and mortality [18]. Recently, Fang et al. [50] found that the Geriatric Nutritional Risk Index can predict poor outcomes after TJA. We further revealed a strong association between treatment failure and moderate-to-severe malnutrition identified by INSTs in PJI patients during the two-stage exchange arthroplasty. This association may be explained by impaired immune function and increased susceptibility of patients to bacterial infections. Nutritional deficiencies can cause decreased host defenses, including impaired mucosal and skin barrier function, reduced monocyte and macrophage counts, and defects in adaptive immune function [22,51,52]. All of the above can lead to the recurrence of infection or failure to eradicate it. However, we must note that our conclusions are preliminary, and further studies are needed to confirm this potential association. 

Given that malnutrition is common in patients with PJI and is associated with treatment failure, the question remains as to which tool is more appropriate for clinicians to use to identify malnourished PJI patients. Our results suggested that CONUT may have a better potential predictive value, although there was a positive correlation between CONUT, NPS, and NRI. In addition, NPS and NRI showed a stronger relationship with ESR. This might be explained by the fact that different tools contain different parameters or different thresholds for the same parameter. Only albumin was common among the parameters included in the three screening tools. However, in the NPS and CONUT scoring systems, the diagnostic threshold for malnutrition is 40 g/L and 35 g/L, respectively, while it is used as a continuous variable in the NRI. As we have seen, the three screening tools showed poor concordance in identifying malnutrition, with prevalence rates ranging from 48.1% to 98.9%, revealing that they are not interchangeable. The above phenomenon has also been demonstrated in studies from other fields [36,37,53]. Moreover, previous studies have indicated that CRP and ESR levels do not perform well in guiding treatment decision making for PJI, suggesting that new alternative indicators are urgently needed [42,54]. Our study preliminarily illustrated the clinical guidance of the CONUT score as a reliable nutritional screening tool. Similarly, CONUT has been demonstrated to have good prognostic value in cancers and other infections [35,55,56]. Although the exact mechanism is uncertain, this association may be explained by the nutritional and immune-related parameters in CONUT [57]. Our results did not indicate an association between mild malnutrition and poor outcome, except for the CONUT score at the 2nd stage. One possible explanation is that there may be differences in the severity of the infection, as the risk of malnutrition increases with the severity of the disease [58]. Furthermore, the RCS model demonstrated that patients with worse nutritional status would be at a higher risk of failure. These findings still need to be confirmed in further studies with large samples. 

Another significant implication of this research is that it could help identify patients at risk for moderate-to-severe malnutrition. The current consensus is that older adults are susceptible to nutritional problems and eventually to malnutrition through various mechanisms [59]. Our results further highlight the significance of nutritional assessment in the elderly population to determine a more beneficial treatment strategy. A recent study demonstrated that a history of previous surgery is associated with a high risk of revision after primary total knee arthroplasty [60]. The meta-analysis also indicated that a history of previous joint surgery was a patient-related risk factor for PJI after TJA [61]. The impact of an increased history of joint surgery and worse nutritional status may be interactive and result in a poor outcome. Overall, we recommend a detailed examination of nutritional status in high-risk patients to further improve their prognosis. 

Previous studies have confirmed that malnutrition plays a detrimental role in different populations [62,63,64]. However, the pathophysiological alterations in malnourished individuals are still not fully elucidated. Regarding adaptive immunity, some studies have shown that malnourished patients with respiratory or gastrointestinal infections have significantly reduced B-cell counts. Peripheral CD8+ and CD4+ T-cell numbers were unchanged in malnourished children with bacterial infections, but some memory T-cell subsets appeared to be reduced [65]. Fasting for as short as two days similarly reduced the number of T cells in the spleen [66]. More importantly, there is significant T-cell depletion in the immune microenvironment of PJI patients with chronic PJI [67], potentially exacerbating the vicious cycle of infection and malnutrition. Future studies are recommended to reveal the altered immune system in malnourished PJI patients so as to explore new therapeutic avenues. 

Several limitations were considered in our study. First, the study design was retrospective, with a limited number of patients enrolled, and certain biases were inherent. We may be able to conduct larger sample size studies in the future and allow for stratified analyses to improve the robustness of the results and explore the impact of different factors on the results. Second, despite the execution of standard institutional treatment guidelines, patient management variation persists over 10 years. Third, only nutritional status at baseline was available, and no indicators were dynamically observed during follow-up. Due to the lack of knowledge about the patient’s diet, there are unknowns related to the effects of macro- and micronutrients as well as dietary patterns on the progression and treatment of PJI. Additionally, the identification of malnutrition was based on definitions, and the exploration of thresholds for each INST score may further strengthen our conclusions. Although ESR and CRP are the most commonly used indicators in PJI diagnosis and reimplantation studies, future exploration of additional inflammatory indicators may provide more information on the nutritional and systemic inflammatory status. Finally, the identification of malnutrition is exclusively dependent on blood parameters and does not allow for the assessment of other conditions that may lead to immune compromise, such as overnutrition. Direct measurement of body composition or scoring questionnaire-based screening, such as the Nutritional Risk Screening 2002 and Malnutrition Universal Screening Tool, may provide new insights into this issue. 

Overall, the significance of the present study is to report the alarming prevalence of malnutrition in patients with PJI and to explore the significant association of malnutrition with their treatment failure for the first time, which means that we have a great scope to improve their prognosis. Given the dramatic increase in the incidence of PJI, we strongly recommend routine nutritional assessment for patients with PJI. Additionally, we highlighted the need for further research to develop targeted interventions and improve clinical outcomes in this vulnerable population. 

## 5. Conclusions

Overall, the prevalence of malnutrition in PJI patients is very high, even exceeding that in some common cancers. Moderate-to-severe malnutrition identified by CONUT at either stage is associated with treatment failure. In addition, advanced age and increased number of previous surgery increase the risk of moderate-to-severe malnutrition. We strongly recommend routine nutritional screening and improving malnutrition in PJI patients, especially those at high risk. 

## Figures and Tables

**Table 1:** Baseline characteristics of the study population at 1st stage exchange. Values are presented as mean ± SD or number of subjects (percentage).

Characteristics	Value
**Demographic Details**	
Age, years	59.6 ± 13.2
Male gender	87 (47.5)
BMI, kg/m^2^	25.0 ± 4.1
Smoking	11 (6.0)
Comorbidities	
Cardiovascular disease	39 (21.3)
Diabetes	24 (13.1)
**Disease Data**	
Hip	104 (56.8)
Primary diagnosis	
Osteoarthritis	52 (28.4)
ONFH	44 (24.0)
DDH	3 (1.6)
Inflammatory arthropathy	11 (6.0)
Fracture	47 (25.7)
Others	26 (14.2)
Pathogens	
*Staphylococcus aureus*	17 (9.3)
*Coagulase-negative Staphylococcus*	52 (28.4)
*Entoococcus*	3 (1.6)
*Streptococcus*	5 (2.7)
*Gram-Negative Bacteria*	9 (4.9)
Others	13 (7.1)
Mixed	30 (16.4)
Negative	50 (29.5)
Number of prior procedures	1.2 ± 1.1
**Laboratory Test**	
Serum albumin, g/L	38.5 ± 3.7
Lymphocyte count, 10^9^/L	1.8 ± 0.6
Total cholesterol, mmol/L	165.9 ± 38.6
C-reactive protein, mg/dL	2.4 ± 2.6
ESR, mm/h	40.7 ± 26.8

ONFH, osteonecrosis of the femoral head; DDH, developmental dysplasia of the hip; ESR, erythrocyte sedimentation rate.

**Table 2:** Correlation of nutritional scores with indicators of systemic inflammation or other nutritional scores.

Indicator	CRP	ESR	CONUT	NPS	NRI
**CONUT**	r = 0.34, *p* < 0.001	r = 0.16, *p* = 0.029		r = 0.63, *p* < 0.001	r = −0.52, *p* < 0.001
**NPS**	r = 0.34, *p* < 0.001	r = 0.24, *p* = 0.001	r = 0.63, *p* < 0.001		r = −0.53, *p* < 0.001
**NRI**	r = −0.29, *p* < 0.001	r = −0.31, *p* < 0.001	r = −0.52, *p* < 0.001	r = −0.53, *p* < 0.001	

CRP, C-reactive protein; ESR, erythrocyte sedimentation rate; CONUT, Controlling Nutritional Status; NRI, Nutritional Risk Index; NPS, Naples Prognostic Score.

**Table 3:** Description of clinical outcomes using the MSIS outcome reporting tool at different starting points of treatment assessment. The numbers in parentheses represent the percentage of people in that tier.

MSIS Outcome	1st Stage Exchange (*n* = 183)	2nd Stage Exchange (*n* = 167)
Tier 1/2	130 (71.0)	130 (77.8)
Tier 3	31 (16.9)	18 (10.8)
Tier 3A	4 (2.2)	4 (2.4)
Tier 3B	2 (1.1)	2 (1.2)
Tier 3C	3 (1.6)	3 (1.8)
Tier 3D	6 (3.3)	6 (3.6)
Tier 3E	3 (1.6)	3 (1.8)
Tier 3F	13 (7.1)	-
Tier 4	22 (12.0)	19 (11.4)
Tier 4A	5 (2.7)	2 (1.2)
Tier 4B	17 (9.3)	17 (10.2)

MSIS, Musculoskeletal Infection Society. Tiers 3 and 4 were defined as total failures, where tiers 3B, 3C, 3D, 3E, 3F, and 4A were failures directly related to PJI, and tiers 3A and 4B were failures due to secondary causes.

**Table 4:** The association between malnutrition and treatment failure due to direct causes.

Definition		Failure Rate	OR * (95% CI)	*p*
**Malnutrition at 1st Stage**				
**CONUT**	Normal	13/95 (13.7%)	Reference Category	
	Mild	13/73 (17.8%)	1.07 (0.38–3.01)	0.891
	Moderate-to-severe	6/15 (40.0%)	5.86 (1.39–24.67)	**0.016**
**NPS**	Normal	2/22 (9.1%)	Reference Category	
	Mild	12/91 (13.2%)	1.06 (0.18–6.14)	0.945
	Moderate-to-severe	18/70 (25.7%)	2.32 (0.41–12.96)	0.239
**NRI**	Normal	13/81 (16.0%)	Reference Category	
	Mild	4/30 (13.3%)	0.55 (0.13–2.30)	0.415
	Moderate-to-severe	15/72 (20.8%)	0.86 (0.27–2.15)	0.603
**Malnutrition at 2nd Stage**				
**CONUT**	Normal	7/111 (6.3%)	Reference Category	
	Mild	7/49 (14.3%)	3.86 (1.03–14.44)	**0.045**
	Moderate-to-severe	2/7 (28.6%)	12.15 (1.14–129.36)	**0.039**
**NPS**	Normal	1/40 (2.5%)	Reference Category	
	Mild	10/96 (10.4%)	11.26 (0.92–138.22)	0.068
	Moderate-to-severe	5/31 (16.1%)	14.39 (1.27–266.22)	**0.033**
**NRI**	Normal	8/115 (7.0%)	Reference Category	
	Mild	2/20 (10.0%)	1.94 (0.28–13.41)	0.501
	Moderate-to-severe	6/32 (18.8%)	3.60 (0.84–15.36)	0.083

CONUT, Controlling Nutritional Status; NRI, Nutritional Risk Index; NPS, Naples Prognostic Score; OR, odds ratio; CI, confidence interval; * Adjusted for baseline characteristics such as age, gender, Body Mass Index (BMI), pathogens, number of previous surgeries, knee or hip joints, primary diagnosis, smoking, and comorbidities.

**Table 5:** Predictors of moderate to severe malnutrition identified by CONUT at the 1st stage exchange.

Variables	Odds Ratio	95% CI	*p*
Age ≥ 68	5.35	1.17–24.52	**0.031**
Female gender	0.31	0.07–1.26	0.101
Number of previous surgeries	2.04	1.20–3.50	**0.009**
Primary diagnosis			
Osteoarthritis	Reference Category		
ONFH	0.11	0.01–0.87	**0.037**
Inflammatory arthropathy	7.16	0.51–101.45	0.146
Fracture	0.17	0.02–1.29	0.087
Others	1.41	0.20–9.86	0.729

CI, confidence interval; ONFH, osteonecrosis of the femoral head. Bold numbers represent *p*-values less than 0.05.

## Data Availability

The data underlying this article cannot be shared publicly due for the privacy of individuals that participated in the study. The data may be shared on reasonable request to the corresponding author.

## Appendix A

**Table A1:** Evolution of patient nutritional status in the two-stage exchange.

Definition	1st Stage Exchange	2nd Stage Exchange	*p*
**CONUT**	1.8 ± 1.7	1.3 ± 1.4	**<0.001**
**NPS**	2.1 ± 1.3	1.4 ± 1.1	**<0.001**
**NRI**	99.3 ± 6.1	102 ± 6.3	**<0.001**

CONUT, Controlling Nutritional Status; NRI, Nutritional Risk Index; NPS, Naples Prognostic Score. Bold numbers represent *p*-values less than 0.05.

## Appendix B

**Table A2:** Association between total treatment failure and malnutrition identified by Controlling Nutritional Status (CONUT).

Definition		Failure Rate	OR (95% CI)	*p*
**Malnutrition at 1st Stage**				
**CONUT**	Normal	28/95 (29.5%)	Reference Category	
	Mild	18/73 (24.6%)	0.72 (0.30−1.76)	0.472
	Moderate-to-severe	7/15 (46.7%)	2.96 (0.75−11.67)	0.102
**Malnutrition at 2nd Stage**				
**CONUT**	Normal	20/111 (17.1%)	Reference Category	
	Mild	14/49 (28.6%)	3.02 (1.10−8.29)	**0.032**
	Moderate-to-severe	3/7 (42.9%)	10.99 (1.38−87.54)	**0.024**

CONUT, Controlling Nutritional Status. Bold numbers represent *p*-values less than 0.05.
